# Digital Services Landscape in Primary Care Setting in City of Zagreb; an EIP-AHA Reference Site Case Study

**Published:** 2019-01-06

**Authors:** V Lazic, N Pjevac, A Masic, L Milutinovic, D Sijak, A Balenovic

**Affiliations:** 1 Health center Zagreb – Center, Zagreb, Croatia

**Keywords:** EIP on AHA, digital services, case study, eHealth, mHealth, telemedicine

## Abstract

European Innovation Partnership on Active and Healthy Ageing (EIP on AHA) brings together partners to create innovative solutions to the challenges of aging. Reference Sites (RS) of the Partnership act as hubs of innovation and assist the scale-up of identified solutions. “Blueprint on Digital Transformation of Health and Care for the Ageing Society” (The Blueprint) is guiding the shift towards ICT enabled patient-centered care. To further inform its development, a tool has been created and piloted across RS, to explore the digital services landscape and find services that address the needs of the personas developed for this tool, that represent the needs of the populations. The aim of this case study was to explore the digital services ecosystem in primary care in Zagreb from the services availability and accessibility perspective, using the personas needs tool. The total of 23 digital services was identified out of which 21 matched at least one persona need. Each service-need match was scored against usefulness and accessibility criteria and the resulting matrix was evaluated using original methods. The results point to several underperforming services and provide insight into possible improvement strategies. Several “workhorse” services were identified that are heavily dependent on the health workforce. The services adopted through EIP on AHA twinning schemes performed well against set criteria. The persona based tool, along with the original service assessment methodology based on the tool’s framework provides a new perspective to the digital services landscape, useful for planning the areas for improvement and detecting underperforming services on a system level.

## I. INTRODUCTION

European Innovation Partnership on Active and Healthy Ageing (EIP on AHA) is an initiative of the European Commission (EC) which has been designed to bring together relevant stakeholders from national and regional levels and from different policy areas to create innovative solutions to the societal challenges that arise from the demographic development of the European population [1,2]

The aim of the partnership is to improve health and quality of life of the aging European citizens, increase the sustainability of the health and social care systems and stimulate economic growth based on the principles of the silver economy [3].

The operational framework of the partnership includes the formation of Reference Sites (RS)- regional partnerships that include stakeholders from healthcare, industry, academia and government on regional level, to act as hubs of innovation, and assist the scale-up of identified solutions, based on the new paradigm of health and care [4]. In December 2016, 74 European regions were awarded the status of RS. The actions of RS should be guided by the shared policy vision, the “Blueprint on Digital Transformation of Health and Care for the Ageing Society”(The Blueprint) [5], which has been endorsed by the EC.

In order to inform the further development of The Blueprint, a new tool has been developed by WE4AHA project [6], based on personas representing different age groups and different levels of complexity of health needs, which reflect the needs of the population (Persona’s needs survey). 12 different personas were invented and divided into 3 groups. A young girl’s Rose, working woman Leila, and elderly Randolph and Theresa belong to “Generally well” group. In the second group, “Chronic conditions & social needs,” the users of the tool are introduced to Millie, a young adult, Nikos, a middle-aged man, and elderly Eleni and Maria. Members of the third group “Complex needs”, are: Ben, a boy with Down’s syndrome, Antonio, paralyzed young adult, and elderly Procolo and Jacqueline. The tool is intended for use “on the ground” to assist the development of innovative patient-centered solutions, identify digital services already in place and screen for potential gaps in the digital services ecosystem.

Health center Zagreb – Center (HCZC) is a public health care provider in the City of Zagreb, Croatia. HCZC provides services of primary care and secondary care level to the population of about 350 000 inhabitants of City of Zagreb and neighboring regions and is the largest health center in Croatia. HCZC has been a lead partner of the City of Zagreb RS (Zagreb RS) of the EIP on AHA since the region was awarded this status in December 2016. Zagreb RS was a beneficiary of the 2017 “Transfer of Innovation Twinning Support Scheme [7],” a part of the ScaleAHA project supported by the EC. RS Zagreb participated in the twinning activities as adopting region for the elements of the Andalusian eHealth Strategy “Diraya.” and the Galician “IANUS” regional EHR system and ePrescription solution. The transferred service elements have been integrated into the existing primary care digital service ecosystem of HCZC in form of new services “Zdravlje.net: Health diary”, “Zdravlje.net: Group messages” and “Zdravlje.net PRO”.

The aim of this study is to explore the digital services ecosystem in primary care in Zagreb from the availability of the services and accessibility perspective, using the Persona’s needs survey, in order to evaluate the existing services, provide insight into potential areas of improvement of existing services, and inform the development of new digital services in primary care, better suited to the needs of the patients.

## II. METHODOLOGY

For this case study, a persona based tool [5] developed by WE4AHA project was used to map the digital services ecosystem in primary care in HCZC. Original methods, based on the framework of the persona tool, were used to further assess the services of the digital landscape in HCZC.

The tool consists of:

- An overview poster with 12 personas grouped according to their needs (generally well/good wellbeing, chronic conditions and/or social needs and complex needs) and to their life course (children/young people, working-age adults, retired people, people aged 80+) in a way that each needs group is represented in one life course stage
- 12 individual persona posters
- Instructions sheet
- Needs summary sheet
- Recording spreadsheet.

Each persona poster consists of 5 major sections for this purpose contextualized by the authors as “Introduction,” “Digital skills,” “Personal story,” ”Overview,” and “Needs.” Introduction section contains the cartoon image of the persona face, name, age, life course (stage), needs (complexity level), connectivity (use of the internet, mobile devices etc.), country and area (urban, suburban, rural). Digital skills section shows the following skills of the persona on a visual scale low to high: Internet usage, Mobile device skills, Affinity to new tech, Digital Health Literacy, Assistance (ICT use). Context section containing a brief “background” story about persona, including personal details, feelings, responsibilities, needs, problems. Overview section contains 8 subsections: What’s important to *name of persona*,” “Daily living,” “Own resources & assets/support,” “Events, issues & personal concerns,” “Health concerns,” “Health tests,” “Treatment: medications, therapies etc.,” “Health professional concerns.” Needs section contains 3–5 listed needs of the persona. These needs are again listed in “needs summary sheet” and appear on the x-axis of the recording spreadsheet.

A three-step protocol was developed and used to complete the recording spreadsheet:

In step 1, a team of 4 reviewers from HCZC listed the available digital services in HCZC on the y-axis of the spreadsheet. Some services were grouped reflecting their similarities in the way they are accessed or used. Reviewers then matched the services with the persona needs in the x-axis. For each match, an explanation was recorded in the predetermined field of the spreadsheet.

In step 2, two independent reviewers, each blind to result of the other, scored each match against two criteria- usefulness to the persona (three-point Likert scale 1 being “barely addresses the patient needs” 2 being “partially addresses the patient needs” 3 being “fully addresses the patient need”) and accessibility to the persona (three-point Likert scale 1 being “barely accessible to the patient”, 2 being “partially accessible to the patient” and 3 being “fully accessible to the patient”).

In step 3, a reviewer consensus was reached on all unequally scored items.

**Figure 2:**
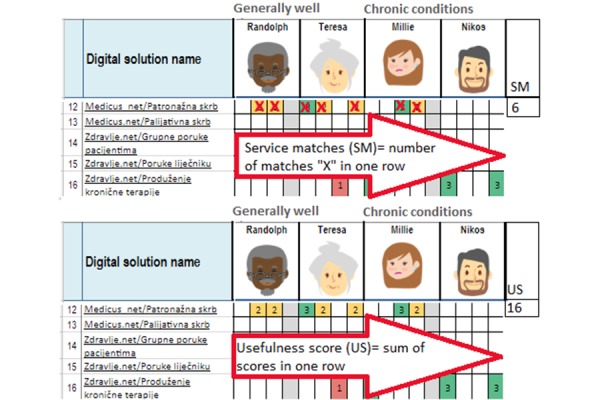
Calculation of SM and US values

A number of needs (out of 49 total persona needs) that each service matched (service matches, SM) and the sum of usefulness scores of those matches across one service (usefulness score, US) was calculated. Figure 2.

Alignment measure (AM) of the service towards complexity groups is US value split into three parts, according to the scores obtained from each complexity group.

Average of accessibility scores (accessibility average, AA) was calculated for each service.

## III. RESULTS

Digital Services availability and digital service performance measures

Reviewers identified 23 digital services available in HCZC. Table 1.

On average, services in “Panel” group matched 3 needs overall (avgSM=3, min=0, max=6). The patient panel dedicated to chronic management of dermatitis failed to meet any of the described persona needs and COPD, OAT and NRS panels (#6,8 and 10) met only a single need. In this group, the highest scoring (US=8) was the diabetes panel (#4) and the pharmacotherapy management chronic patient panel (#11). None of the services from this group scored a “3” on usefulness scale for any of the persona needs.

The #12 Medicus.net- Field nursing. *(SM=21, US=42, AM=18/14/10, AA=2)* was the highest scoring service, with both SM and US as the highest (example, persona Leila, need 2- *eAppointment for a field nurse visit to conduct insulin administration training)*. The service was more aligned to the needs of the generally well population with AM 18/14/10 spread across complexity groups. AA was 2 for this service.

Palliative care as a service, providing a structured communication channel between GP and Palliative care team, aimed at improving the coordination of care of palliative and end-of-life patients did not match to any of the persona needs.

Zdravlje.net group, consisting of services numbered 14 to 19, was the highest scoring group overall, with high SM, US and AA values across service range *(SM=5, US=8, AM=2/4/2, AA=3)*

The highest scoring was #15 Patient-GP messaging and #19 Appointment scheduling services. The lowest scorings were #14 Patient group messaging and #18 Health Diary: Hypertension. The AM differed across the group. AA of Zdravlje.net group was the highest overall.

Zdravlje.net PRO group, enabling GP consultation with other specialists and counseling services, showed an average SM with the high US, and AM towards chronic and complex needs. AA was 2 for both services.

eAppointment showed above average SM and relatively high US. AM of this service was “U” shaped (10/5/14), similar to the shape of that of service #15. AA was 2.

ePrescription showed low SM and US. AM was aligned towards more complex needs. AA was 3.

## IV. DISCUSSION

Based on the used methodology, new insight was created into the complexity of the existing digital services in HCZC. The persona based tool, along with the original service assessment methodology based on the tool’s framework provides a crude overview of the performance of services.

Field nursing (#12), GP-patient messaging (#15), and GP appointment scheduling (#19) scored the highest both in SM and in the US with the latter two also having a high AA (3 and 2,8). These are conceptualized by the authors as “workhorse” services, providing a starting point in the further development of the digital service based system. The newest additions in the digital service repertoire of HCZC, the services adopted from the ScaleAHA twinning, “Zdravlje.net: Health diary” (#17,18), “Zdravlje.net: Group messages” (#14) and “Zdravlje.net PRO” (#20,21) blended exceptionally well into the service matrix with Zdravlje.net PRO group services being among the highest scoring overall.

The “Panel” group of services (#1–11), underperformed on all measures indicating a need to change the concept of physician-based chronic condition monitoring. As the performance of these services has never been evaluated in a formal way and taking into account the reported increase in chronic disease morbidity and mortality in Croatia, these results could point to an important gap in healthcare provision. In order to increase the SM of these services, they would need to be redesigned to a more patient-based form. If the right to view and edit various panel values was extended from GP to the patients, this could create an interesting platform for exchange, similar to the much higher scored “Health diary”. Additional improvements could also include integrated message and image sharing, task and goal setting and progress tracking.

The high scoring Field nursing and GP-patient messaging services (#12 and 15) point to a problem of high dependency of newly developed digital services on the health workforce. Thus, the lack of patient- and community-based services leads to an increasing burden on already stretched-out healthcare workforce, all in the context of population aging and increasing medical and social needs. The abundance of services with little usefulness in terms of not addressing any of the patients needs to the problem of lack of strategic planning of digital service development, underdevelopment of the digital service market and poor innovation management, lacking real co-development and patient involvement elements.

Due to the largely qualitative nature of this case study potential sources of bias include, non exclusively: inherent design bias of the used tool (e.g. over-representation of a certain type of persona need could have influenced the downstream process and reasoning leading to faulty conclusions), procedural bias in the step 2 reviewer group leading to over-matching and low service usefulness scores, confirmation and culture bias might have led the authors to misinterpret the findings. Potential pitfalls in the results interpretation (e.g. low SM and US scores could indicate both low performance and high focus of service) should be avoided through careful evaluation.

## V. CONCLUSION

The persona based tool, along with the original service assessment methodology based on the tool’s framework, although lacking in finesse, can provide a new perspective of the digital services landscape, useful for planning the areas for improvement and detecting underperforming services on a system level. Shifting focus to individual needs instead of individual services might produce more interesting insights into the functioning of the services matrix, as this would allow the analysis of multiple service synergies.

## 



## Figures and Tables

**Figure 1:**
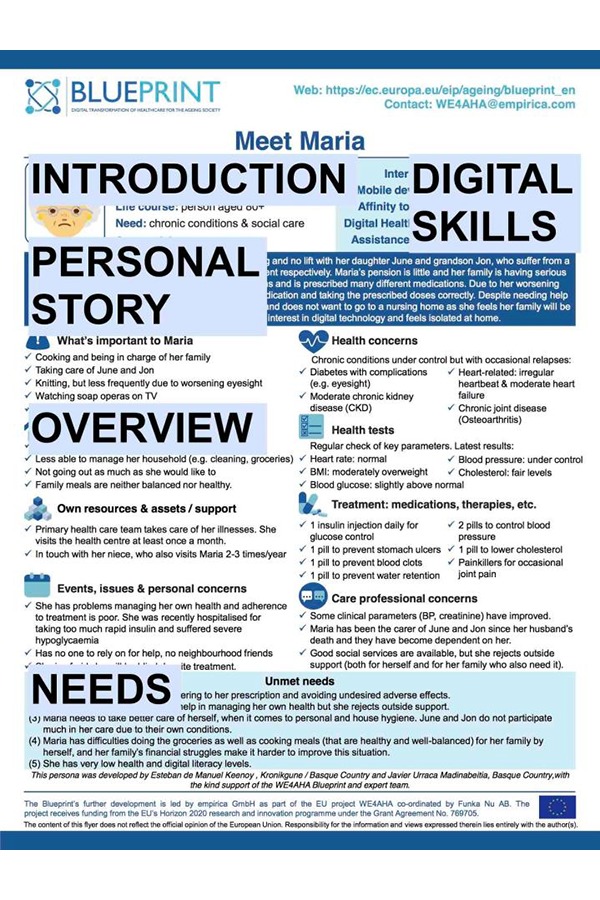
Example persona poster with highlighted sections * This persona was developed by Esteban de Manuel Keenoy, Kronikgune/Basque Country and Javier Urraca Madinabeitia, Basque Country, with the support of the WE4AHA Blueprint and expert team, as part of the EU project WE4AHA (H2020 RIA Grant Agreement No.769705)

**Table 1:** Digital services of HCZC list with an example of addressed persona need together with selected performance measures of HCZC digital services

#	Digital Service name	TYPICAL use case [Persona#need]	SM***	US	AM1	AM2	AM3	AA
1	Panel- Child development	Growth and nurture status panel enables GP* to monitor growth and development in children from preschool-age	2	2	1	0	1	2.0
2	Panel- Dermatitis**	Dermatitis panel enables the GP to collect relevant information about patients new/old skin changes, monitoring the development of changes and the efficiency of administered treatment.	0	0	0	0	0	-
3	Panel- Prevention	Panel “Prevention” enables GP to evaluate patients without chronic conditions. In case of an appearance of pathological value, the system alarms the GP to react with preventive measures.	5	6	1	3	2	2.0
4	Panel- Diabetes	Diabetes panels enable the GP to evaluate and keep track of the severity of existent diabetes mellitus and preventing comorbidities.	6	8	0	4	4	2.0
5	Panel- Hypertension	Hypertension panel enables GP to measure factors related to appearance, development and worsening of arterial hypertension	2	4	0	0	4	2.0
6	Panel- COPD	COPD panel enables the GP to evaluate the clinical status of the patient with beforehand diagnosed lung disease: Asthma, Emphysema, Chronic bronchitis. Panel directions the doctor to carry out needed therapeutic measures	1	2	0	2	0	2.0
7	Panel- Body mass index (BMI)	BMI panel serves to GP as a tool for estimation of patients physical condition. Besides calculating body mass index, the system uses other needed information to estimate the risk of chronic diseases development. In the case of pathological values and answers consistent with an unhealthy way of life, the system warns the GP to undertake preventive measures and to fill out related panels	5	5	1	1	3	2.0
8	Panel- Nutritional risk assessment	Nutritional status panel enables GP to monitor weight, BMI, physical activity, habits of the patient.	1	2	0	2	0	2.0
9	Panel- Cardiovascular risk	Cardiovascular risk panel enables GP to asses the risk of developing cardiovascular disease	4	5	0	2	3	2.0
10	Panel- Oral anticoagulant therapy	Facilitates monitoring and provision of oral anticoagulant therapy for GP and facilitates adherence to therapy for patients	1	1	0	1	0	2.0
11	Panel- Pharmacotherapy	Pharmacotherapy panel enables the GP to: get insight in patients’ way of taking medication, patient’s understanding of the importance of taking the medication as prescribed into patients’ compliance Enables the GP to call the patient for a check-up with instructions to bring all medication with him Panel enables the GP to give patient exact written instructions of taking his prescribed therapy	6	8	0	3	5	2.0
12	Medicus.net- Field nursing	Two-way communication between the GP and field nurses based on an application.	21	42	18	14	10	2.0
13	Medicus.net- Palliative care**	Two-way communication app for GP- mobile palliative team communication	0	0	0	0	0	-
14	Zdravlje.net- Patient group messaging	Group messaging app enables the GP to send out the same message to a larger number of patients. GP can select a patient by sex, diagnosis.	5	8	2	4	2	3.0
15	Zdravlje.net- GP-patient messaging	Direct message based secure channel for GP-patient communication	19	37	13	10	14	3.0
16	Zdravlje.cet- Chronic therapy request	The patient app that enables sending a request for ePrescription renewal for chronic therapy	7	16	1	11	4	2.9
17	Zdravlje.net- Health diary: Diabetes	Patient leads a “Health diary” in an app and data is available to GP at any time. Diabetes section is used for monitoring blood glucose values. The system evaluates the values and puts them in categories: low, normal, elevated, and warns the patient and the GP.	7	16	0	9	7	3.0
18	Zdravlje.net: Health Diary: Hypertension	Patient leads a “Health diary” in an app and data is available to GP at any time. Hypertension section includes blood pressure and pulse values. The system evaluates the values and puts them in categories: low, normal, elevated, and warns the patient and the GP	4	9	0	5	4	2.8
19	Zdravlje.net- GP Appointment scheduling	The application enables the patient to make appointments in the GP’s office	19	40	13	14	13	2.8
20	Zdravlje.netPRO/eConsultation GP-other specialist	eConsulting is designed to create a direct contact between a GP and a specialist in primary or secondary health care.	12	28	6	8	14	2.0
21	Zdravlje net PRO/eConsultation GP-counselling service	eConsulting is designed to create a direct contact between a GP and counseling centers for nutrition or pharmacotherapy	9	27	3	12	12	2.0
22	eAppointment scheduling for secondary care	Enables the GP to make an appointment for a patient for a specialist checkup or diagnostics	12	29	10	5	14	2.0
23	ePrescription	ePrescription system: prescription information is provided by the GP, stored in the national system and available on request to a pharmacy.	6	14	2	7	5	3.0

* General practitioner

** Solutions 2 and 13 did not match any of the persona needs

*** SM-service matches, US- usefulness score, AM1/2/3- alignment measure for groups 1(generally well), 2(chronic conditions & social needs) and 3 (complex needs). AA-average accessibility score. Color range code- red 1^st^ percentile, yellow 50^th^ percentile, the green 99^th^ percentile
