# Supplementary material for: Digital Services Landscape in Primary Care Setting in City of Zagreb; an EIP-AHA Reference Site Case Study
Source: Transl Med UniSa. 2019 Jan 6;19:124–8. (PMC6581491)
Supplement: Supplementary file 1 [file TM-19-124-s001.doc]

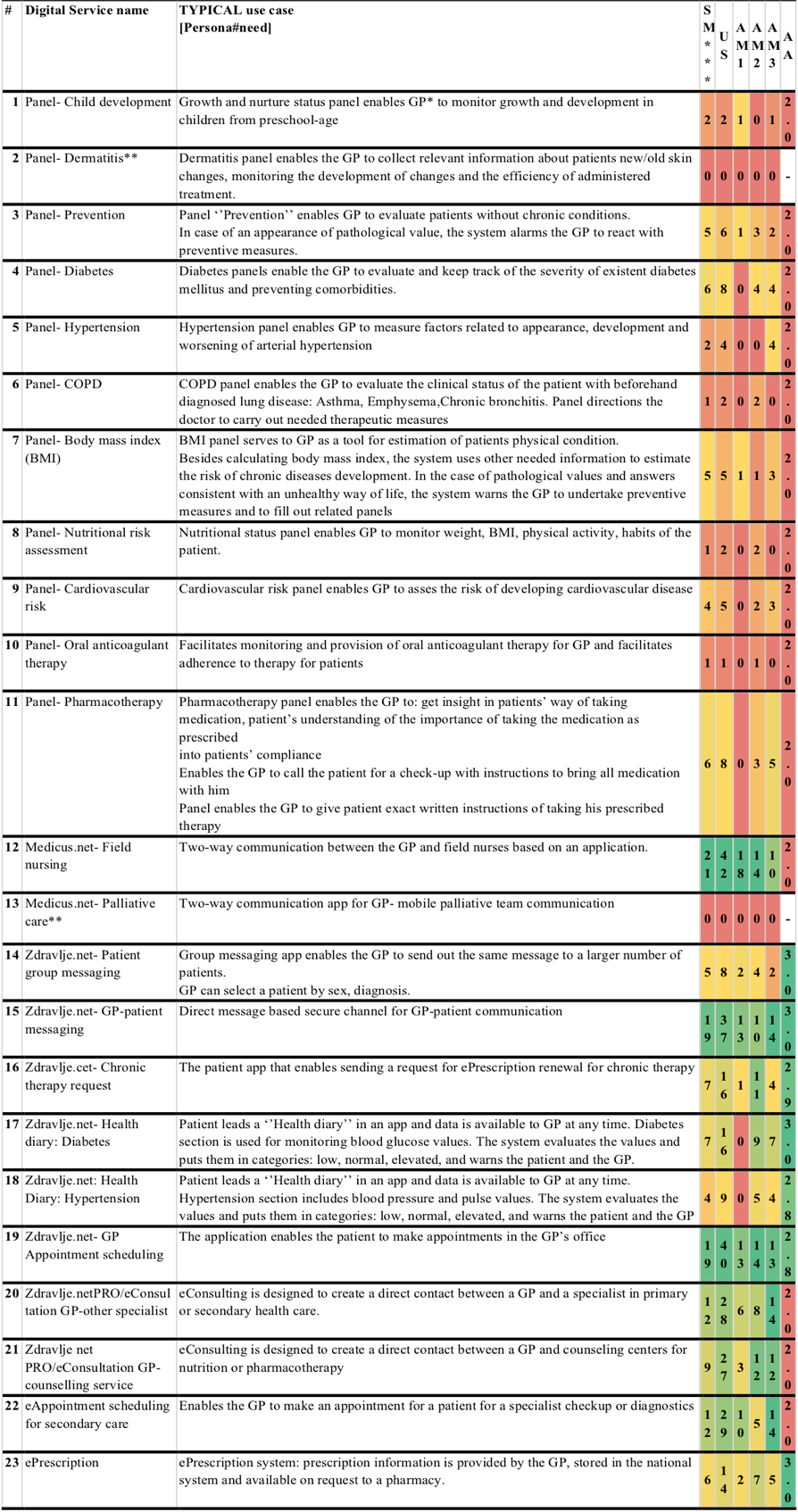
**Table 1: Digital services of HCZC list with an example of addressed persona need together with selected performance measures of HCZC digital services**

**General practitioner*

***Solutions 2 and 13 did not match any of the persona needs*

****SM-service matches, US- usefulness score, AM1/2/3- alignment measure for groups 1(generally well), 2(chronic conditions & social needs) and 3 (complex needs). AA-average accessibility score. Color range code- red 1^st^ percentile, yellow 50^th^ percentile, the green 99^th^ percentile*
